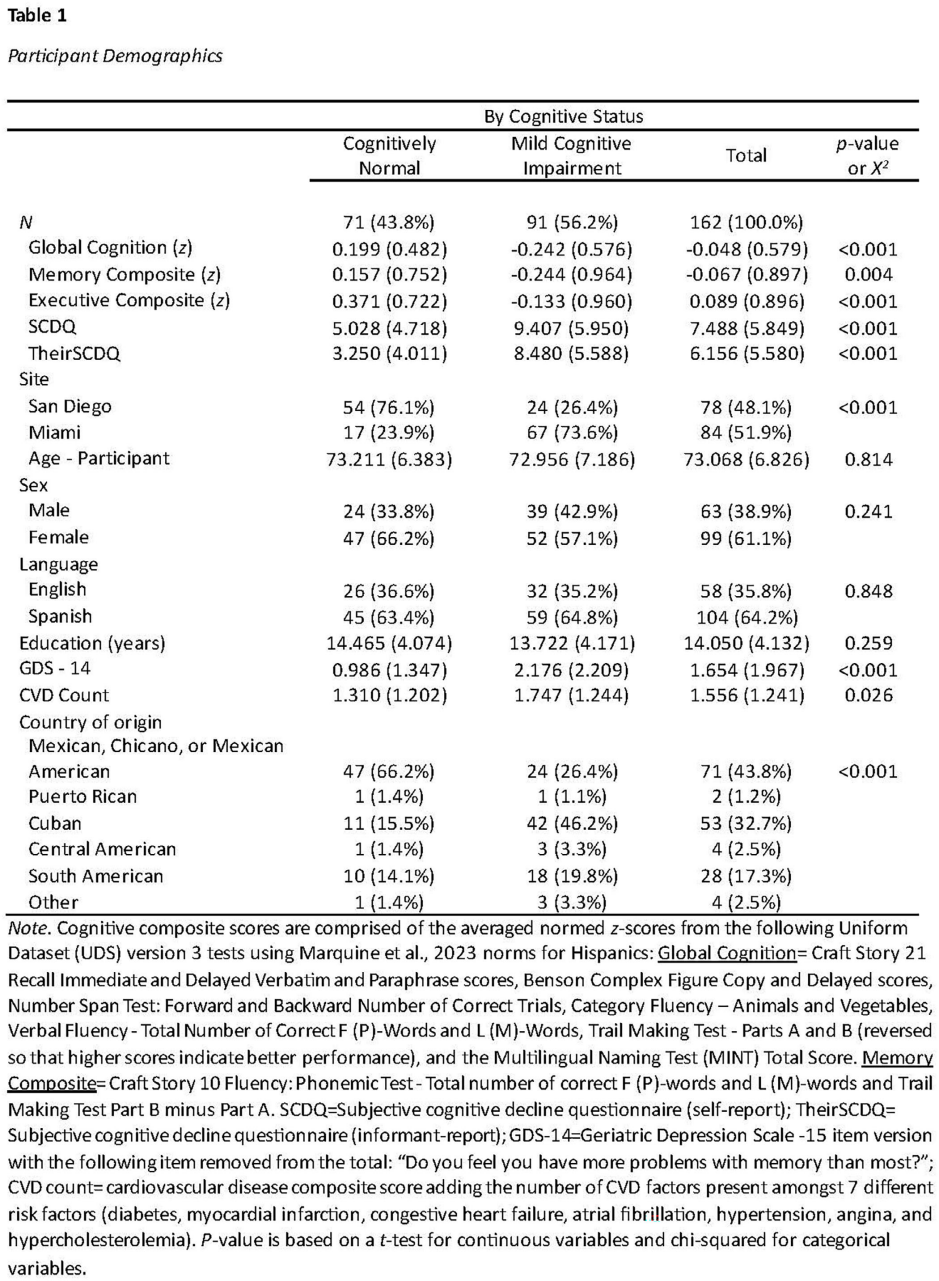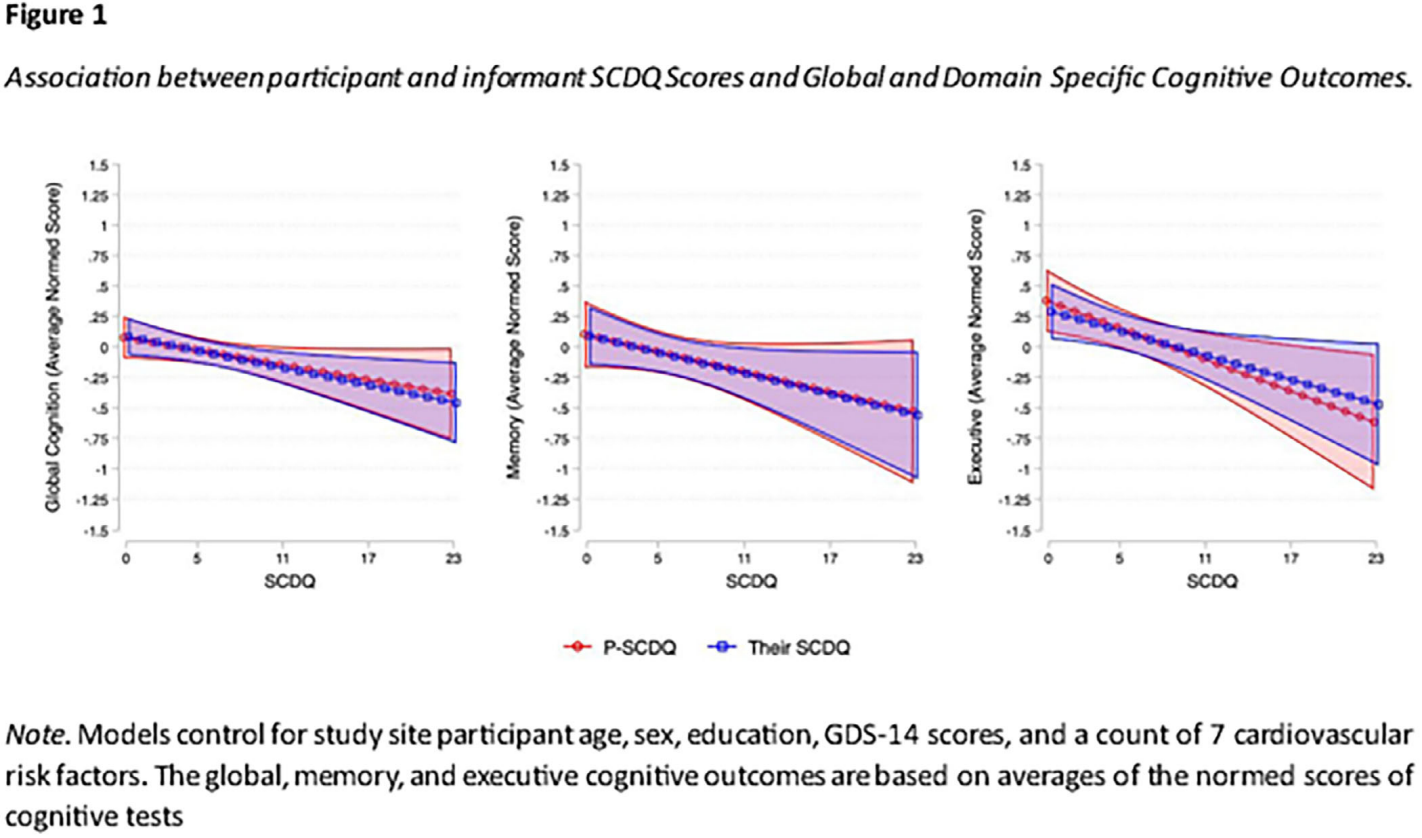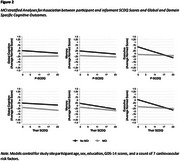# Subjective cognitive decline severity and objective cognition in a diverse a sample of Hispanic adults from two Alzheimer's disease research centers

**DOI:** 10.1002/alz70857_106625

**Published:** 2025-12-26

**Authors:** Yaimara Gonzalez Pinero, Wassim Tarraf, Idaly Velez‐Uribe, Warren W Barker, Michelle Villar, David P. Salmon, Douglas R. Galasko, Ranjan Duara, Maria J. Marquine, Zvinka Z. Zlatar

**Affiliations:** ^1^ 1 Florida Alzheimer's Disease Research Center (ADRC), Miami Beach, FL, USA; ^2^ Wien Center for Alzheimer's Disease and Memory Disorders at Mount Sinai Medical Center, Miami Beach, FL, USA; ^3^ Wayne State University, Detroit, MI, USA; ^4^ Wien Center for Alzheimer's Disease and Memory Disorders, Miami Beach, FL, USA; ^5^ Mount Sinai Medical Center, Miami Beach, FL, USA; ^6^ Florida Atlantic University, Davie, FL, USA; ^7^ 1Florida Alzheimer's Disease Research Center, Miami, FL, USA; ^8^ University of California, San Diego, La Jolla, CA, USA; ^9^ Duke University School of Medicine, Durham, NC, USA

## Abstract

**Background:**

Self‐perceived or informant‐reported subjective cognitive decline (SCD) may be an early predictor of objective cognitive decline and Alzheimer's disease. However, little is known about its clinical validity and utility in underrepresented populations. This study investigated if the severity of self‐ or informant‐reported SCD is associated with objective cognition in a diverse sample of Hispanic older adults.

**Methods:**

We recruited 162 Hispanics without dementia (*M_age_* =  73 years, *SD* = 6.8; *M_edu_* = 14 years, *SD* = 4.1; 61.1% women; 64.2% tested in Spanish) and 135 informants, from the UC San Diego Shiley‐Marcos Alzheimer's Disease Research Center (ADRC) and the 1Florida ADRC (Miami, FL). The self (SCDQ) and informant (TheirSCDQ) versions of the SCD Questionnaire and the objective cognitive tests from the Uniform Data Set version 3 (UDS3) were administered. Linear regression analyses investigated associations of SCD severity (SCDQ and TheirSCDQ) with global and domain‐specific (memory and executive) normed UDS3 performance while adjusting for site, demographics, depression, and cardiovascular disease risk factors. Analyses were repeated stratifying by cognitive status (i.e., cognitively normal [CN] vs. mild cognitive impairment [MCI]), since those with MCI may not accurately report SCD and informant reports may be more clinically useful.

**Results:**

In fully adjusted models, SCDQ significantly predicted executive function (*b* = ‐0.03, *CI* = [‐0.06; ‐0.01], *p* = < .05) while TheirSCDQ predicted global cognition (*b* = ‐0.0, *CI* = [‐0.04; ‐0.01], *p* < .05), memory performance (*b* = ‐0.03, *CI* = [‐0.06; ‐0.00], *p* < .05), and executive function (*b* = ‐0.03, *CI* = [‐0.06; ‐0.01], *p* < .05). Analyses stratified by cognitive status suggested that associations between SCD severity and objective cognition were driven by CN individuals and not by those with MCI (descriptively, as the sample size did not allow for testing interactions).

**Conclusion:**

In this sample of diverse Hispanic older adults, severity of self‐ and informant‐reported SCD is associated with objective cognitive test scores, with informant‐report associated with more cognitive domains than self‐report. Findings support the potential clinical utility of SCD measures in Hispanic older adults, but further studies are needed to determine if SCD predicts longitudinal changes in cognitive performance.